# A Rapid Extraction Method for mammalian cell cultures, suitable for quantitative immunoblotting analysis of proteins, including phosphorylated GCN2 and eIF2α

**DOI:** 10.1016/j.mex.2017.10.008

**Published:** 2017-10-28

**Authors:** Richard C. Silva, Beatriz A. Castilho, Evelyn Sattlegger

**Affiliations:** a Department of Microbiology, Immunology and Parasitology, Escola Paulista de Medicina, Universidade Federal de São Paulo, São Paulo 04023-062, Brazil; b Institute of Natural and Mathematical Sciences, Massey University, Auckland 0745, New Zealand

**Keywords:** Rapid Protein Extraction Method (RPE method), eIF2α, α subunit of translation initiation factor 2, GCN2, General Control non-derepressible 2, PBS, phosphate-buffered saline, SDS-PAGE, SDS polyacrylamide gel electrophoresis, Mammalian cell culture, cell lysis, SDS-PAGE, quantitation, western blot, GCN2, eIF2 alpha

## Abstract

Many studies require the detection and relative quantitation of proteins from cell culture samples using immunoblotting. Limiting factors are the cost of protease inhibitors, the time required to break cells and generate samples, as well as the high risk of protein loss during cell breakage procedures. In addition, a common problem is the viscosity of lysed samples due to the released genomic DNA. As a consequence, the DNA needs to be broken down prior to denaturing polyacrylamide protein gel electrophoresis (SDS-PAGE), e.g. by passing the sample through a syringe gauge needle, sonication, or DNase treatment. In a quest to find a more cost-effective, fast, and yet robust procedure, we found that cell lysis, protein denaturation, and DNA fragmentation can be done in only two steps: harvesting followed by a simple non-laborious 2nd step. Similarly to many pre-existing cell breakage procedures, in our Rapid Protein Extraction (RPE) method, proteins liberated from cells are immediately exposed to a denaturing environment. However, advantages of our method are:

• No breaking buffer is needed, instead proteins are liberated directly into the denaturing protein loading buffer used for SDS-PAGE. Consequently, our RPE method does not require any expensive inhibitors.
• The RPE method does not involve post-lysis centrifugation steps; instead all cell material is dissolved during the 2nd step, the mixing-heat-treatment step which is new to this method. This prevents potential protein loss that may occur during centrifugation. In addition, this 2nd step simultaneously shears the genomic DNA, making an additional step for DNA fragmentation unnecessary.
• The generated samples are suitable for high-quality quantitative immunoblotting. With our RPE method we successfully quantified the phosphorylated forms of protein kinase GCN2 and its substrate eIF2α. In fact, the western signals were stronger and with less background, as compared to samples generated with a pre-existing method.

## Graphical abstract

**Figure:**
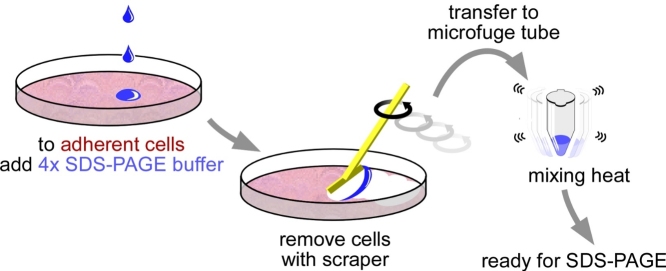

## Method details

### Materials

#### Reagents

• Cell lines cultured in 6 cm diameter round plastic dishes (0.5–1 × 10^6^ cells) or 6-well microtiter plates (0.25–0.5 × 10^6^ cells), ready for harvesting
• 6 cm diameter round plastic dishes, Greiner Bio-One, # 628160, or TPP, # 93060
• 6-well microtiter plates, Nunc, Thermo Fisher Scientific, # 140675, or TPP, # 92406
• NaCl Ajax Finechem, Thermo Fisher Scientific, # AJA465
• KCl, Ajax Finechem, Thermo Fisher Scientific, # AJA383
• Na_2_HPO_4_, Sigma-Aldrich, # S5136
• KH_2_PO_4_, Sigma-Aldrich, # P5379
• Tris, Formedium, UK, # TRIS01
• HCl to adjust pH, Ajax Finechem, Thermo Fisher Scientific, # AJA1
• Glycerol, Ajax Finechem, Thermo Fisher Scientific, # AJA242
• Sodium dodecyl sulfate (SDS), Sigma-Aldrich, # L3771
• 2-mercaptoethanol (14.3 M), Sigma-Aldrich, # M6250
• Bromophenol blue Sigma-Aldrich, # B5525
• Ice chips
• Dry ice or liquid nitrogen are preferred, otherwise use:
• Metal rack able to hold 1.5 ml tubes, precooled in −20 °C or −80 °C freezer.
  e.g. 80-well Chamber for 1.5 ml tubes, Diversified Biotech, USA, # CHAM-8000, or CoolRack
  Thermoconductive Tube Racks, BioCision # M30-PF, also sold by Sigma-Aldrich, # BCS-128

#### Recipes

• *Denaturing Protein Sample Buffer*:
  0,625M Tris-HCl pH 6,8; 10% glycerol; 3% SDS; 0.5 mM EDTA, 5% (v/v) 2-mercaptoetanol, 0,1% (w/v) bromophenol blue, distribute 1 ml in 1.5 ml tubes, freeze. Recipe adapted from [3]. One difference to the published recipe is that the Tris-HCl concentration is higher to increase the buffering capacity of the buffer, however, we did not test whether this is critical for this method.
• *Phosphate-buffered saline (PBS)*:
  8 g/L (127 mM) NaCl, 0.2 g/L (2.7 mM) KCl, 1.44 g/L (10 mM) Na_2_HPO_4_, 0.24 g/L (1.8 mM) KH_2_PO_4_, adjust to pH 7.4 with HCl

#### Equipment

• Aspirator, e.g. Bel-Art Products, Thomas Scientific, # 1185B57:
  or otherwise Pipett aid with 10 and 5 ml serological pipettes
• 1.5 ml microfuge tubes, one per sample
• 1.5 ml microfuge tubes, for aliquoting Denaturing Protein Sample Buffer
• 20–200 μl pipette, yellow tips
• Cell scraper, with angled swivel blade:
  length ∼1.3 cm (for 6-well microtiter plate) or 2 cm (for 6 cm plastic dish).
  e.g. Corning # 07-200-365 (1.8 cm), or TPP, # 99002 (1.3 cm) or # 99003 (2 cm)

• Table top microtube centrifuge, 4 °C and room temperature (RT)
• −80 °C (preferred) or −20 °C freezer
• Shaking heating block, such as Digital Shaking Drybath, Thermo Fisher Scientific, # 8880028, or Eppendorff Thermomixer C-PF-19703, or Heat/Cool Thermal Mixer, Boekel, # 270811

## Procedure

Step 1: Harvesting cells

**Fig. 1:**
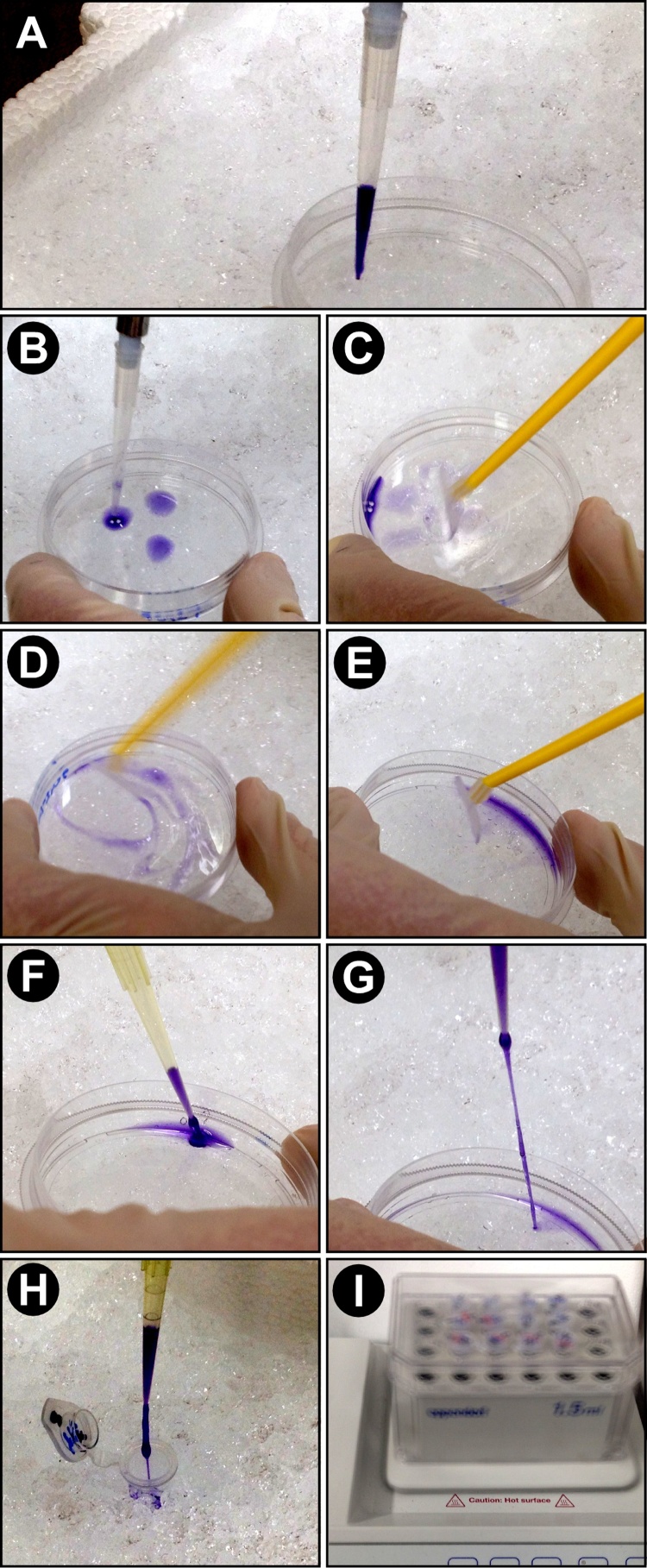
Harvesting cells from plates during the Rapid Protein Extraction (RPE) Method. One plate at a time was removed from the incubator and placed on ice. After removal of the medium from the plate, the adherent cells were washed with ice cold PBS. A) Denaturing Protein Sample Buffer was taken up with a pipette and B) transferred onto the plate. C) A spatula was placed in the plate and D) with circular motions all cells were detached from the plate. The buffer became viscous, indicative of cells being detached and starting to lyse. E) With the spatula the sample was collected on one side of the plate and F) using a 200 μl pipette the sample was taken up slowly. G) The entire sample was retrieved before moving the pipette tip away from the plate. In this image, the tip was moved away from the plate prematurely, to illustrate the viscosity of the sample. H) The sample was transferred to a 4 °C cold microfuge tube, and optimally transferred directly to the bottom of the tube (Not done here in order to better illustrate the slimy appearance of the sample). Samples were then immediately frozen (not shown). I) For complete protein denaturation necessary for resolving proteins by size in SDS-PAGE gels, and for shearing the genomic DNA, the frozen samples were directly placed onto a 99 °C shaking heating block for 10 min, mixing at maximum speed. After this treatment, samples had fully lost their viscous behaviour.
  add 80 μl Denaturing Protein Sample Buffer to the middle of the plate (Fig. 1B).
8) Immediately, using a cell scraper (∼3 cm long blade for 6 cm plastic dish), remove cells from the plastic surface (Fig. 1C). For this, with one hand press the spatula onto the plastic surface of the dish. While keeping the angle of the blade the same, move the entire scraper in a circular motion, following a circle that ranges from the rim of the plate into 2/3 of the plate’s diameter. At the same time, slowly rotate the plate clockwise, by 360°. Repeat, but rotate plate counter-clockwise.
  During this procedure the liquid becomes viscous (Fig. 1D).
9) Holding the plate in an angle, use the scraper to collect the entire sample in the lowest part of the plate (Fig. 1E).
10) Using a 200 μl pipette, transfer sample to the bottom of a 1.5 ml microfuge tube. Pipet slowly as sample is viscous (Fig. 1F–H).
11) If sample is not in the bottom of the tube, quickly spin in a centrifuge for a few seconds, 4 °C, ∼1000 g.
12) Immediately place sample on dry ice or in liquid nitrogen. If not available, transfer tube to a −80 °C or −20 °C cold metal block, to quickly freeze the sample.

1) Seed cells into 6 cm diameter plastic dishes, and conduct experiment as planned.
2) Prior to harvesting, thaw Denaturing Protein Sample Buffer at room temperature.
  Ensure that the SDS becomes fully dissolved.
3) At time of harvesting: Remove one plate at a time from the incubator, and place on ice.
4) Holding the plate in an angle, remove as much medium as possible, using an aspirator.
5) Level the plate, add 4 ml ice-cold PBS, swirl plate quickly.
6) Holding the plate in an angle, remove as much liquid as possible, using an aspirator.
7) Level the plate (Fig. 1A):

Step 2: Mixing Heat treatment

13) Equilibrate shaking heating block to 99 °C.
14) Transfer frozen samples directly to shaking heating block.
15) Incubate in mixing heating block for 10 min, at 99 °C, with maximum rpm (Fig. 1I).

The samples are ready for denaturing gel electrophoresis. The amount of sample to be loaded on the gel depends on the abundance of the protein of interest, and may require optimization for each specific protein. See anticipated results for directions.

If not used immediately, samples can be stored at −20 °C or −80 °C until analysis. Thaw and mix samples well prior to loading.

## Anticipated results

The anticipated outcome is the obtention of mammalian cell culture protein extracts suitable for quantitative western blot analysis. These extracts are generated via a cost-effective and quick lysis procedure that does not require a breaking buffer nor inhibitors, but still provide conditions that significantly reduce the risk of protein degradation and modifications that may occur during harvesting and cell lysis. In this Rapid Protein Extraction (RPE) Method, in step 1, cells were harvested by directly scraping them from the surface of the culture vessel in the presence of a Denaturing Protein Sample Buffer suitable for denaturing polyacrylamide protein gel electrophoresis (PAGE). During this procedure the sample buffer became viscous, indicative of efficient cell lysis, and the sample was easily transferrable to a microfuge tube. Step 2 arose from our discovery that heating samples at 99 °C for 10 min in a shaking heating block, fully removed the viscosity of the sample. This indicated that in addition to protein denaturation, the high temperature in combination with shaking in the presence of SDS has also sufficiently sheared the DNA. Notably, western blots generated from such samples were of high quality and quantifiable. In particular, the phosphorylated form of the protein GCN2 (GCN2-P) – usually hard to detect with conventional methods (e.g. [[1], [2]]) – was readily detectable in cell extracts generated via the RPE Method. Our method does not allow for the determination of the total protein amount in the sample. However, this seems to be unnecessary given that one can probe for housekeeping proteins, such as GAPDH or actin, to normalise for equal loading. In addition, the cell density at the time of harvesting can be controlled, and we observed that this method of cell breakage hardly leads to any sample loss, making it relatively easy to load similar total protein amounts. Approximately 0.5–1 × 10^6^ cells were harvested in 80 μl of final sample volume. This method was optimised for adherent cells, but could also be applicable for cells in suspension.

In order to test our procedure and its suitability for western blotting, in particular for scoring the amount of phospho-proteins, we aimed to detect the phosphorylation levels of the lowly abundant protein kinase GCN2 and its substrate eIF2α. GCN2 is best known for its function in monitoring amino acid availability in cells. When it detects an amino acid shortage, it auto-phosphorylates, and then phosphorylates eIF2α. Elevated eIF2α-P levels activate a signaling pathway that ultimately leads to a reprogramming of the cell’s gene expression profile, to allow cells to cope with and overcome starvation [4].

**Fig. 2:**
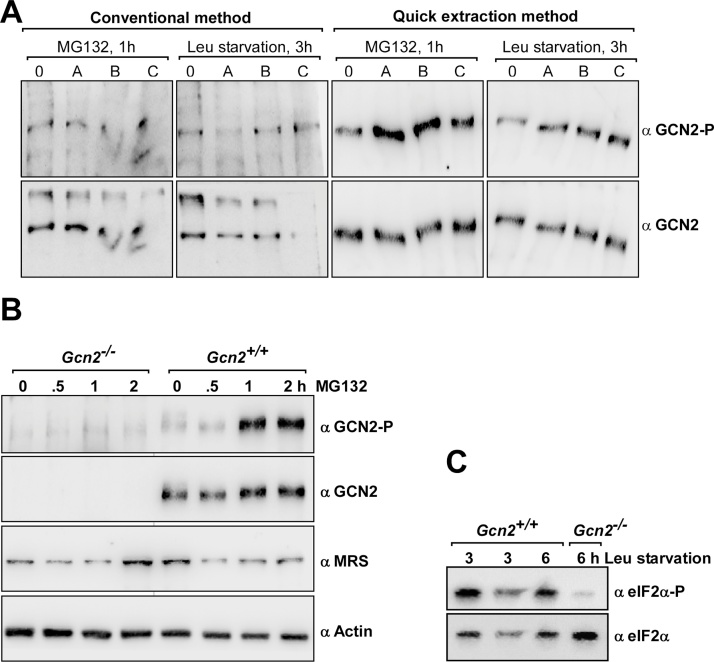
Western blot results using samples generated via the Rapid Protein Extraction (RPE) Method. A) MEFs were grown in cell culture flasks to ≤80% confluence in high glucose DMEM medium (Gibco Thermo Fisher Scientific, # 11965-092), supplemented with sodium pyruvate (1:100, 100 mM, Gibco Thermo Fisher Scientific, # 11360-070), penicillin and streptomycin antibiotic mix (1:100, 10,000 U/Ml, PenStrep, Gibco Thermo Fisher Scientific, # 15140-122), and fetal bovine serum (FBS) (1:10, heat inactivated at 60 °C for 1 h, Gibco Thermo Fisher Scientific, # 10270106). The medium was removed, the cells washed with PBS, detached from the flasks via a 5 min treatment with 0.25% trypsin (Gibco Thermo Fisher Scientific, # 15050-057), and then counted in a hemocytometer. 5 × 10^5^ MEFs were seeded into 6 cm diameter plastic dishes in 4 ml of the above medium. The next day, the medium was removed, the dish washed with 4 ml PBS, followed by the addition of 4 ml DMEM medium lacking Leu (Vitrocell Embriolife, Brazil, # 00367, lacking Gln, Pyr and Leu), supplemented with Pyruvate, Gln (1:100, 200 mM, Gibco Thermo Fisher Scientific, # 25030-081), and dialysed FBS (1:10, heat inactivated as above, Gibco Thermo Fisher Scientific, # 26400-044) (three independent samples labelled A, B, C), or the same medium but containing Leu (Vitrocell Embriolife, Brazil, # 02209) (sample labelled as 0), or DMEM medium containing 2 μM MG132 (Sigma Aldrich, #M7449, diluted in DMSO) (three independent samples, labelled A, B, C). After the indicated incubation time, cells were subjected to the RPE Method. 20 μl of each sample was subjected to SDS-PAGE using a 6% denaturing acrylamide gel, and then the proteins transferred to a nitrocellulose membrane (Hybond-C Extra 0.45 μm, Amersham GE Healthcare Life Sciences, # RPN303E, or 0.2 μm Nitrocellulose membrane, Bio-Rad, # 1620112) via the wet transfer method (Mini Trans-Blot Electrophoretic Transfer Cell, Bio-Rad), at 100 V for 1 h in Tris Glycine buffer (25 mM Tris base, 192 mM glycine) containing 0.1% SDS. The membrane was subjected to immunoblotting, as described in Fig. 3, using antibodies against the phosphorylated form of Gcn2 (GCN2-P. 1:1000, Abcam, # ab75836). Thereafter, the membrane was treated with 15% H_2_O_2_ (30% H_2_O_2_ diluted in H_2_O, Ajax Finechem, Thermo Fisher Scientific, # 260) for 30 min (modified from [8]), extensively washed, and probed with antibodies against GCN2 [9]. B) Gcn2^+/+^ and Gcn2^−/−^ MEFs were grown and samples generated as above, subjected to SDS-PAGE using a 6% gel, and to immunoblotting using antibodies against Gcn2, Gcn2-P, actin (1:5000, Sigma-Aldrich, # A2066), and Methionyl-tRNA Synthetase (MRS, 1:2000, Abcam, # ab50793). C) Samples were generated as above, and 5 μl of each sample subjected to SDS-PAGE using a 10% gel, and to immunoblotting using antibodies against eIF2α (1:1000, Thermo Fisher Scientific, # AHO0802), the phosphorylated form of eIF2α (eIF2α-P, 1:2000, Thermo Fisher Scientific, # 44-728G). eIF2α-P was detected first, the membrane treated with H_2_O_2_ as outlined in A) and then probed for eIF2α.

GCN2-P is sometimes hard to detect in western blots of cell extracts (see below), making it difficult to score for Gcn2-P levels. To determine the suitability of the RPE Method for detecting GCN2-P, we grew Gcn2^+/+^ and Gcn2^−/−^ mouse embryonic fibroblast cells (MEFs) in Dulbeccós Modified Eaglés medium (DMEM) as described previously [5]. The next day, plates were washed with phosphate buffered saline (PBS), and then subjected for 3 h to DMEM lacking L-leucine (Leu) to trigger Leu-starvation, or for 1 h to DMEM containing 2 μM of the proteasome inhibitor MG132, known to stimulate GCN2 activity [6]. Cells were harvested following the above RPE Method. In parallel, an identical set of samples was treated similarly but then subjected to one of the currently used harvesting procedures as done in [2]. Then, the same fraction of all samples was subjected side-by-side to SDS-PAGE using a 6% denaturing polyacrylamide gel, and to immunoblotting. We found that samples generated with the pre-existing method produced a band of the expected size of GCN2, but also an additional unspecific band (Fig. 2A). However, samples prepared with the RPE Method only showed the Gcn2 signal, even after prolonged exposure times (not shown). Notably, the Gcn2 signal was stronger in samples generated with the RPE Method as compared to the pre-existing method. Both, the pre-existing as well as the RPE Method release proteins into a denaturing environment, however, the stronger signal with the RPE method may be due to the fact that after cell breakage no cell debris – that may potentially contain proteins of interest – is removed, but instead is brought back into solution. Notably, the RPE Method allowed a much clearer detection of GCN2-P. When probing for Gcn2-P, the samples prepared via the RPE method generated a far stronger signal in westerns, as compared to the ones prepared via the pre-existing method, and at the same time the background noise was considerably weaker.

We also tested our RPE Method for western blots using various antibodies, such as, for example, antibodies against actin and the Methionine tRNA Synthetase. For this, Gcn2^+/+^ and Gcn2^−/−^ MEFs were grown as above, but exposed to MG132 for 0.5, 1, and 2 h. As before, the GCN2 antibodies generated a clear signal in the samples obtained from Gcn2^+/+^ cells, and it was clearly visible that GCN2-P increased 1–2 h after MG132 treatment (Fig. 2B). Actin and the Methionine tRNA Synthetase could also be readily detected.

Next, we tested for the detection of eIF2α and eIF2α-P. Gcn2^+/+^ and Gcn2^−/−^ MEFs were grown and subjected to Leu starvation, as above. The bands for eIF2α and eIF2α-P were readily detectable, and the low eIF2α-P levels in the Gcn2^−/−^ MEFs were clearly visible as well (Fig. 2C).

**Fig. 3:**
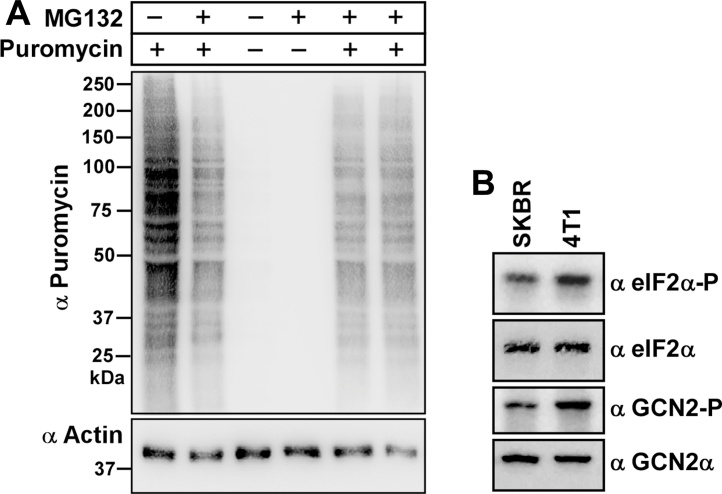
Further testing of the Rapid Protein Extraction (RPE) Method. A) Gcn2^+/+^ MEF cells were grown in medium with or without 2 μM MG132 for 1 h, and then incubated in medium containing puromycin for 10 min prior to harvesting. 10 μl of samples were subjected to immunoblotting with anti-puromycin antibodies (1:5000, Millipore-MABE343, clone 12D10) [7] and, after stripping [10], with anti-actin antibodies, as described in [5]. Molecular markers are depicted in kDa on the left side of the image. B) 5 × 10^5^ human (SKBR3) or mouse (4T1) breast cancer cell lines were each grown overnight in 6 cm plastic dishes containing 4 ml RPMI 1640 medium (Gibco Thermo Fisher Scientific, # 72400-047), supplemented with FBS, sodium pyruvate and antibiotics as above. After subjecting cells to the RPE method, 20 μl and 5 μl of the cell extract were resolved via SDS-PAGE in 6% (for detecting GCN2 and Gcn2-P, 20 μl equate to ∼50 μg of total protein loaded) and 10% (eIF2α and eIF2α-P, 5 μl equate to ∼12.5 μg of total protein loaded) denaturing Tris acrylamide gels, followed by immunoblotting to detect the indicated proteins. Working solutions containing primary antibodies were diluted in standard Tris buffered saline containing 5% (w/v) skim milk powder, or 3% (w/v) BSA (for eIF2α-P antibodies, BSA fraction-V IgG-free, Gibco Thermo Fisher Scientific, # 30063721). Primary antibodies were detected with horseradish peroxidase (HRP) conjugated to goat anti-rabbit antibodies (1:50,000, Pierce, Thermo Fisher Scientific, # 31460; for the detection of actin, MRS, Gcn2-P and eIF2α-P antibodies), goat anti-guinea pig (1:5000, Santa Cruz, # SC-2020; Gcn2), or to goat anti-mouse antibodies (1:50,000, Pierce, Thermo Fisher Scientific, # 31430; actin, eIF2α, puromycin). HRP was visualized using a chemiluminescence detection solution (SuperSignal West Pico Chemiluminescent Substrate, Pierce, Thermo Fisher Scientific, # 34080, or Lumina Forte Western HRP substrate, Merck Millipore, # WBLUF0100) and the Alliance 4.7 transilluminator (UVITEC Limited, Cambridge, UK).

In another study we tested our method on samples originated from an *in vivo* translation assay. Puromycin exposure leads to cells incorporating this compound into proteins during translation, and these proteins can be detected with antibodies directed against puromycin [7]. Hence, the higher the translation rate in cells, the stronger the bands detected by these antibodies. This assay was conducted as outlined in [5], and the cells were harvested and lysed using the RPE Method. The western blot was probed for actin to show that the sample amounts loaded were similar (Fig. 3A). The results nicely showed the expected banding pattern in samples treated with puromycin, while the intensity of the bands was decreased in presence of MG132, indicative of impaired translation.

Finally, we validated that the RPE Method is also applicable to cell lines other than MEFs. Accordingly, we grew mouse and human breast cancer cell lines 4T1 and SKBR3 in Roswell Park Memorial Institute (RPMI) medium, followed by harvesting, lysis, SDS-PAGE and western blotting. We found that eIF2α and eIF2α-P, and GCN2 and GCN2-P, can be readily detected (Fig. 3B). Signal exposure times were under 1 min for all the 4 proteins, as found for extracts generated from MEFs (see above), as well as from mouse Neuro 2a cells (N2a, mouse neuroblastoma cell line, data not shown).

Taken together, our studies strongly suggest that the RPE Method is robust, and applicable for the quantitative detection of a large range of proteins in various cell lines.
